# Supplementary material for: Effects of Epiphytes and Depth on Seagrass Spectral Profiles: Case Study of Gulf St. Vincent, South Australia
Source: Int J Environ Res Public Health. 2019 Jul 29;16(15):2701. doi: 10.3390/ijerph16152701 (PMC6696022; doi:10.3390/ijerph16152701)
Supplement: Supplementary file 1 [file ijerph-16-02701-s001.pdf]

Supplementary 1. Non-significant wavelengths at which the reflectance for seagrass leaf tops do not differ from that of leaf sheaths/stem bases.

wavelength (nm)	p-value	Reflectance, leaf top	Reflectance, stem base
670	8.7503E-02	0.020509351	0.020170142
671	8.8497E-02	0.020087183	0.019848747
672	7.2076E-01	0.018968223	0.019282719
673	3.8893E-01	0.018504292	0.019082964
674	4.9859E-01	0.018227019	0.018707995
675	9.3728E-01	0.018321237	0.018418424
676	7.3664E-01	0.018190574	0.018171312
677	8.9175E-02	0.017975805	0.018219639
678	2.2598E-01	0.01815209	0.018450451
679	3.2651E-01	0.017926377	0.018292834

.

Supplementary 2. Statistically significant wavelengths at which reflectance for leaf sheath/stem base generally tended to be higher in value than that of leaf top.

wavelength (nm)	p-value	Reflectance, leaf top	Reflectance, stem base
350	5.6E-03	1.8431E-03	1.1553E-03
351	7.5E-03	1.7193E-03	1.2439E-03
352	2.2E-03	1.8010E-03	1.2825E-03
353	1.2E-05	1.9496E-03	1.2862E-03
354	5.4E-06	2.1490E-03	1.4253E-03
355	3.3E-05	2.2396E-03	1.6192E-03
356	1.8E-06	2.2125E-03	1.6529E-03
357	2.0E-05	2.0838E-03	1.5138E-03
358	7.2E-03	1.7499E-03	1.3431E-03
359	3.1E-02	1.7393E-03	1.3926E-03
360	1.1E-03	1.9310E-03	1.4276E-03
361	1.8E-05	2.1669E-03	1.5154E-03
362	2.8E-05	2.1707E-03	1.5325E-03
363	1.3E-04	2.1273E-03	1.5396E-03
364	2.0E-04	2.2633E-03	1.6704E-03
365	1.3E-05	2.3176E-03	1.6325E-03
366	2.8E-07	2.6133E-03	1.8217E-03
367	1.9E-06	2.7562E-03	1.9649E-03
368	3.8E-06	2.7514E-03	1.9799E-03
369	2.6E-05	2.7596E-03	2.0831E-03
370	2.1E-07	2.6936E-03	1.9187E-03
371	2.5E-08	2.7511E-03	1.9488E-03
372	5.9E-05	2.7541E-03	2.0980E-03
373	2.3E-07	2.7696E-03	1.9376E-03
374	2.8E-08	2.5202E-03	1.7242E-03
375	1.2E-07	2.4185E-03	1.6393E-03
376	1.7E-08	2.5828E-03	1.6139E-03
377	1.4E-05	2.8443E-03	2.0814E-03
378	5.4E-08	3.0856E-03	2.2310E-03
379	1.2E-15	3.3080E-03	2.2271E-03
380	2.6E-15	3.3875E-03	2.1839E-03
381	2.0E-11	3.3290E-03	2.1530E-03
382	3.8E-12	3.1608E-03	2.0416E-03
383	1.1E-09	2.7113E-03	1.7581E-03
384	1.0E-03	2.2166E-03	1.7415E-03
385	1.8E-05	2.5703E-03	1.8400E-03
386	1.4E-07	2.9718E-03	2.1094E-03
387	6.0E-07	2.9436E-03	2.1891E-03
388	1.6E-07	2.8534E-03	2.1268E-03
389	2.8E-09	2.9068E-03	2.0962E-03
390	6.3E-10	3.2553E-03	2.3214E-03
391	2.2E-12	3.6444E-03	2.6002E-03
392	5.9E-17	3.7660E-03	2.5897E-03
393	5.6E-21	3.3722E-03	2.3386E-03
394	1.8E-14	2.6823E-03	1.9586E-03
395	5.1E-05	2.8813E-03	2.2343E-03
396	1.9E-09	3.6117E-03	2.6677E-03
397	5.6E-15	3.4131E-03	2.4666E-03
398	1.5E-09	2.9860E-03	2.4227E-03
399	4.2E-10	4.0159E-03	3.1788E-03
400	3.0E-13	5.2049E-03	4.0897E-03
401	2.7E-17	5.9838E-03	4.5102E-03
402	2.0E-26	5.9340E-03	4.4252E-03
403	8.5E-22	6.0500E-03	4.8899E-03
404	3.1E-13	6.5825E-03	5.2939E-03
405	9.6E-14	6.5043E-03	5.2907E-03
406	3.0E-14	6.1760E-03	5.1892E-03
407	1.3E-14	6.2359E-03	5.0314E-03
408	1.7E-14	6.5637E-03	5.1557E-03
409	7.0E-15	7.1740E-03	5.5208E-03
410	1.1E-15	7.1506E-03	5.7494E-03
411	9.0E-17	6.8853E-03	5.6357E-03
412	1.0E-24	7.0906E-03	5.4291E-03
413	4.5E-19	7.9109E-03	6.0810E-03
414	2.9E-15	7.9372E-03	6.3467E-03
415	1.6E-12	8.1879E-03	6.5756E-03
416	4.3E-16	7.9340E-03	6.3478E-03
417	4.5E-14	8.0371E-03	6.5859E-03
418	4.7E-12	8.0237E-03	6.6641E-03
419	7.2E-12	7.7802E-03	6.5299E-03
420	1.9E-09	7.9728E-03	6.7625E-03
421	1.0E-08	7.9662E-03	6.7676E-03
422	1.3E-10	8.3353E-03	6.9110E-03
423	3.0E-14	8.3971E-03	6.7858E-03
424	9.6E-10	7.9310E-03	6.7980E-03
425	4.9E-08	7.9195E-03	7.0070E-03
426	1.8E-08	8.2757E-03	6.9878E-03
427	2.6E-09	8.4493E-03	7.0651E-03
428	4.4E-09	8.4420E-03	7.1541E-03
429	9.9E-11	8.3917E-03	6.9513E-03
430	1.1E-08	8.0939E-03	6.7978E-03
431	1.8E-10	7.3613E-03	6.0406E-03

	432	6.4E-19	7.4261E-03	5.6256E-03	
	433	1.9E-13	8.7442E-03	6.9380E-03	
	434	7.6E-10	9.3422E-03	7.7916E-03	
	435	8.3E-06	9.1207E-03	7.9721E-03	
	436	2.4E-05	9.7371E-03	8.4794E-03	
	437	1.2E-11	1.0452E-02	8.7160E-03	
	438	2.8E-12	1.1007E-02	8.9881E-03	
	439	3.2E-06	1.0533E-02	9.0571E-03	
	440	3.9E-07	1.0377E-02	8.9270E-03	
	441	6.5E-11	1.0815E-02	8.9450E-03	
	442	9.4E-11	1.1391E-02	9.3724E-03	
	443	3.0E-10	1.2259E-02	1.0100E-02	
	444	9.3E-08	1.3282E-02	1.1195E-02	
	445	1.5E-07	1.3493E-02	1.1423E-02	
	446	5.7E-07	1.2901E-02	1.1181E-02	
	447	5.5E-06	1.2440E-02	1.0956E-02	
	448	3.3E-06	1.2784E-02	1.1472E-02	
	449	1.3E-03	1.3538E-02	1.2375E-02	
	450	8.2E-05	1.4709E-02	1.3200E-02	
	451	1.5E-04	1.5297E-02	1.3787E-02	
	452	6.2E-05	1.5508E-02	1.3940E-02	
	453	2.6E-07	1.5355E-02	1.3482E-02	
	454	4.1E-07	1.5290E-02	1.3408E-02	
	455	1.3E-06	1.5468E-02	1.3771E-02	
	456	1.8E-09	1.5833E-02	1.3884E-02	
	457	5.3E-11	1.6539E-02	1.4176E-02	
	458	4.4E-06	1.6652E-02	1.4788E-02	
	459	5.1E-03	1.6454E-02	1.5113E-02	
	460	9.5E-04	1.6630E-02	1.5052E-02	
	461	3.7E-04	1.6523E-02	1.5248E-02	
	462	1.3E-05	1.7344E-02	1.5876E-02	
	463	9.2E-08	1.8075E-02	1.6439E-02	
	464	8.4E-06	1.8293E-02	1.6978E-02	
	465	7.4E-04	1.8177E-02	1.6800E-02	
	466	8.8E-06	1.8409E-02	1.6884E-02	
	467	9.9E-06	1.8902E-02	1.7376E-02	
	468	7.0E-06	1.9517E-02	1.7680E-02	
	469	6.4E-06	2.0272E-02	1.8458E-02	
	470	1.5E-04	2.0482E-02	1.8922E-02	
	471	7.4E-04	2.0232E-02	1.8592E-02	
	472	2.8E-04	2.0953E-02	1.9172E-02	
	473	1.1E-05	2.2790E-02	2.0875E-02	
	474	3.4E-08	2.4394E-02	2.2378E-02	
	475	3.0E-10	2.5267E-02	2.3489E-02	
	476	6.6E-09	2.5731E-02	2.4210E-02	
	477	6.6E-05	2.6167E-02	2.4108E-02	
	478	1.4E-05	2.8042E-02	2.5397E-02	
	479	1.5E-07	3.0395E-02	2.7645E-02	

	480	1.4E-07	3.1632E-02	2.9112E-02	
	481	7.3E-08	3.2179E-02	3.0265E-02	
	482	2.1E-09	3.2854E-02	3.1473E-02	
	483	6.0E-09	3.4498E-02	3.2689E-02	
	484	5.9E-09	3.5332E-02	3.2242E-02	
	485	7.7E-14	3.6018E-02	3.2660E-02	
	486	2.6E-09	3.5109E-02	3.2314E-02	
	487	2.8E-10	3.3138E-02	3.0377E-02	
	488	2.4E-16	3.3262E-02	3.0911E-02	
	489	9.6E-10	3.5073E-02	3.3680E-02	
	490	7.8E-07	3.6636E-02	3.4522E-02	
	491	1.1E-09	3.9213E-02	3.6737E-02	
	492	7.0E-10	4.0673E-02	3.8132E-02	
	493	2.0E-08	3.9570E-02	3.7481E-02	
	494	5.3E-11	3.9800E-02	3.8335E-02	
	495	4.8E-11	4.1521E-02	3.9809E-02	
	496	6.3E-09	4.2373E-02	4.0858E-02	
	497	4.4E-10	4.2917E-02	4.2236E-02	
	498	7.9E-08	4.3942E-02	4.2398E-02	
	499	6.9E-10	4.3605E-02	4.2447E-02	
	500	5.6E-17	4.3402E-02	4.3585E-02	
	501	1.7E-15	4.4420E-02	4.3305E-02	
	502	4.1E-10	4.4031E-02	4.3635E-02	
	503	1.3E-13	4.5022E-02	4.5904E-02	
	504	8.7E-21	4.7284E-02	4.7765E-02	
	505	3.5E-20	4.7407E-02	4.8393E-02	
	506	3.3E-22	4.8849E-02	5.0839E-02	
	507	2.7E-21	5.0489E-02	5.2701E-02	
	508	3.5E-26	5.1954E-02	5.4614E-02	
	509	1.2E-30	5.3382E-02	5.6804E-02	
	510	9.8E-24	5.5595E-02	5.8599E-02	
	511	9.2E-29	5.6958E-02	6.1033E-02	
	512	2.4E-33	5.7880E-02	6.2402E-02	
	513	2.2E-31	5.8165E-02	6.3269E-02	
	514	4.6E-51	5.8819E-02	6.6720E-02	
	515	2.9E-60	6.0238E-02	6.8149E-02	
	516	1.1E-48	6.2859E-02	6.9971E-02	
	517	5.1E-53	6.2741E-02	7.1103E-02	
	518	1.8E-66	6.0227E-02	6.8672E-02	
	519	5.8E-59	6.0550E-02	6.8954E-02	
	520	5.4E-58	6.5122E-02	7.3474E-02	
	521	3.7E-62	6.7921E-02	7.8741E-02	
	522	1.1E-60	7.0613E-02	8.4038E-02	
	523	6.1E-61	7.4350E-02	8.7659E-02	
	524	7.8E-77	7.6058E-02	8.9324E-02	
	525	4.9E-82	7.7076E-02	9.1755E-02	
	526	6.4E-74	7.9231E-02	9.5573E-02	
	527	3.8E-86	7.9812E-02	9.6716E-02	

	528	4.4E-95	7.8575E-02	9.5581E-02	
	529	4.8E-83	8.0517E-02	9.6511E-02	
	530	2.7E-61	8.3373E-02	9.9409E-02	
	531	1.7E-76	8.6638E-02	1.0470E-01	
	532	1.8E-91	8.7777E-02	1.0757E-01	
	533	5.2E-90	8.7512E-02	1.0758E-01	
	534	5.5E-95	8.6465E-02	1.0507E-01	
	535	3.0E-88	8.6100E-02	1.0348E-01	
	536	3.4E-88	8.7050E-02	1.0648E-01	
	537	4.6E-103	8.8301E-02	1.1013E-01	
	538	1.4E-83	8.9047E-02	1.0878E-01	
	539	1.0E-68	8.7495E-02	1.0777E-01	
	540	1.8E-90	8.4290E-02	1.0760E-01	
	541	8.3E-95	8.2840E-02	1.0489E-01	
	542	4.1E-103	8.3673E-02	1.0559E-01	
	543	1.6E-96	8.5629E-02	1.0625E-01	
	544	3.4E-88	8.6525E-02	1.0471E-01	
	545	3.1E-95	8.7517E-02	1.0591E-01	
	546	1.5E-93	8.8224E-02	1.0884E-01	
	547	6.1E-112	8.6546E-02	1.1091E-01	
	548	2.1E-124	8.5692E-02	1.0974E-01	
	549	6.8E-98	8.4554E-02	1.0742E-01	
	550	4.2E-79	8.3459E-02	1.0567E-01	
	551	2.4E-76	8.4804E-02	1.0493E-01	
	552	3.0E-83	8.5939E-02	1.0594E-01	
	553	2.0E-98	8.5651E-02	1.0797E-01	
	554	2.8E-91	8.4134E-02	1.0608E-01	
	555	6.7E-76	8.6380E-02	1.0744E-01	
	556	9.7E-95	8.5614E-02	1.0816E-01	
	557	1.1E-95	8.4250E-02	1.0698E-01	
	558	6.7E-93	8.5303E-02	1.0624E-01	
	559	9.8E-100	8.3170E-02	1.0299E-01	
	560	3.5E-94	7.9505E-02	9.9925E-02	
	561	2.6E-85	8.1068E-02	1.0057E-01	
	562	1.0E-82	8.1463E-02	1.0105E-01	
	563	5.9E-87	7.9881E-02	1.0032E-01	
	564	3.3E-96	8.1496E-02	1.0180E-01	
	565	2.6E-102	8.2004E-02	1.0230E-01	
	566	4.6E-94	8.0582E-02	9.9608E-02	
	567	3.1E-86	7.9244E-02	9.7197E-02	
	568	9.3E-101	7.7612E-02	9.7210E-02	
	569	6.5E-98	7.6717E-02	9.6803E-02	
	570	8.6E-86	7.6242E-02	9.4422E-02	
	571	1.9E-84	7.4389E-02	9.0810E-02	
	572	1.3E-82	7.3799E-02	8.9795E-02	
	573	1.4E-77	7.5303E-02	8.9722E-02	
	574	2.3E-74	7.6851E-02	9.1321E-02	
	575	1.5E-65	7.6509E-02	9.2445E-02	

	576	1.7E-65	7.3863E-02	8.9869E-02	
	577	4.0E-65	7.3106E-02	8.7449E-02	
	578	5.0E-69	7.4443E-02	8.7000E-02	
	579	1.1E-59	7.5092E-02	8.7123E-02	
	580	2.1E-61	7.4583E-02	8.7707E-02	
	581	4.6E-80	7.4085E-02	8.9214E-02	
	582	1.7E-78	7.3975E-02	8.8249E-02	
	583	2.7E-76	7.3510E-02	8.7194E-02	
	584	2.8E-71	7.4539E-02	8.7802E-02	
	585	8.6E-67	7.4676E-02	8.7238E-02	
	586	2.0E-56	7.4079E-02	8.4727E-02	
	587	2.5E-63	7.2229E-02	8.2970E-02	
	588	6.4E-77	7.1209E-02	8.3874E-02	
	589	7.7E-70	7.0118E-02	8.0635E-02	
	590	4.1E-62	6.7958E-02	7.6155E-02	
	591	3.5E-55	6.5705E-02	7.4771E-02	
	592	2.4E-58	6.5709E-02	7.6384E-02	
	593	1.6E-63	6.6652E-02	7.7589E-02	
	594	6.4E-52	6.7798E-02	7.5382E-02	
	595	6.1E-53	6.6154E-02	7.4207E-02	
	596	6.9E-60	6.5308E-02	7.5254E-02	
	597	7.8E-54	6.6630E-02	7.4239E-02	
	598	5.6E-58	6.6383E-02	7.3800E-02	
	599	2.2E-59	6.7064E-02	7.4431E-02	
	600	7.6E-59	6.5689E-02	7.4067E-02	
	601	3.3E-55	6.4098E-02	7.2150E-02	
	602	2.8E-51	6.4005E-02	7.0828E-02	
	603	4.0E-58	6.3608E-02	7.0195E-02	
	604	7.7E-55	6.2115E-02	6.9447E-02	
	605	2.8E-46	6.1265E-02	6.8934E-02	
	606	1.4E-47	6.1770E-02	6.8073E-02	
	607	2.8E-55	6.0971E-02	6.8154E-02	
	608	3.9E-57	5.9604E-02	6.7677E-02	
	609	1.6E-55	5.8610E-02	6.5344E-02	
	610	9.0E-52	5.7056E-02	6.3333E-02	
	611	1.5E-47	5.6148E-02	6.2880E-02	
	612	2.3E-46	5.6447E-02	6.2114E-02	
	613	3.6E-46	5.5937E-02	5.9783E-02	
	614	8.3E-46	5.3932E-02	5.8964E-02	
	615	4.4E-43	5.2691E-02	5.8562E-02	
	616	1.3E-36	5.3432E-02	5.7728E-02	
	617	3.1E-45	5.2609E-02	5.8329E-02	
	618	2.0E-50	5.1438E-02	5.7879E-02	
	619	4.6E-47	5.1863E-02	5.7810E-02	
	620	2.0E-44	5.2059E-02	5.7700E-02	
	621	8.7E-39	5.2317E-02	5.7589E-02	
	622	6.6E-30	5.2729E-02	5.6495E-02	
	623	1.1E-35	5.1637E-02	5.6093E-02	

	624	1.2E-39	5.0105E-02	5.5345E-02	
	625	4.2E-34	4.9274E-02	5.3876E-02	
	626	2.4E-38	4.7732E-02	5.3815E-02	
	627	9.7E-40	4.7991E-02	5.4165E-02	
	628	8.5E-32	4.8349E-02	5.4543E-02	
	629	2.0E-41	4.7431E-02	5.4457E-02	
	630	1.2E-45	4.6972E-02	5.3337E-02	
	631	7.2E-38	4.7574E-02	5.1466E-02	
	632	7.7E-42	4.6854E-02	5.0946E-02	
	633	3.4E-35	4.6690E-02	5.0503E-02	
	634	4.1E-31	4.6399E-02	4.9440E-02	
	635	4.9E-29	4.5702E-02	4.9136E-02	
	636	5.3E-25	4.5128E-02	4.8296E-02	
	637	4.3E-31	4.3728E-02	4.7740E-02	
	638	6.5E-40	4.3938E-02	4.7910E-02	
	639	6.7E-42	4.3311E-02	4.7806E-02	
	640	1.4E-31	4.1641E-02	4.6300E-02	
	641	5.2E-28	4.1341E-02	4.3529E-02	
	642	1.3E-37	4.0688E-02	4.2114E-02	
	643	1.1E-29	3.8811E-02	4.1953E-02	
	644	3.2E-22	3.7934E-02	4.0780E-02	
	645	7.7E-18	3.7562E-02	3.9325E-02	
	646	1.3E-19	3.6728E-02	3.7455E-02	
	647	5.3E-18	3.5916E-02	3.5282E-02	
	648	1.1E-14	3.4670E-02	3.4747E-02	
	649	6.8E-18	3.3346E-02	3.4464E-02	
	650	8.1E-19	3.2287E-02	3.3020E-02	
	651	6.0E-14	3.2121E-02	3.1932E-02	
	652	1.4E-13	3.1268E-02	3.0905E-02	
	653	1.5E-15	3.0500E-02	3.0808E-02	
	654	7.3E-12	3.0528E-02	2.9777E-02	
	655	5.3E-16	2.9980E-02	2.8983E-02	
	656	9.5E-08	2.8336E-02	2.7423E-02	
	657	1.2E-03	2.6260E-02	2.4903E-02	
	658	1.6E-05	2.5419E-02	2.4146E-02	
	659	3.1E-09	2.5895E-02	2.5345E-02	
	660	1.7E-09	2.5833E-02	2.5661E-02	
	661	2.8E-08	2.5394E-02	2.5320E-02	
	662	3.3E-05	2.4890E-02	2.4668E-02	
	663	2.3E-04	2.4510E-02	2.4441E-02	
	664	1.6E-03	2.3711E-02	2.3135E-02	
	665	4.0E-05	2.3509E-02	2.2252E-02	
	666	8.9E-03	2.2394E-02	2.1606E-02	
	667	1.7E-02	2.1729E-02	2.1468E-02	
	668	1.0E-03	2.1173E-02	2.1077E-02	
	669	3.3E-03	2.0773E-02	2.0480E-02	
	680	2.3E-03	1.7625E-02	1.8274E-02	
	681	4.4E-04	1.8282E-02	1.8244E-02	

	682	1.9E-04	1.8365E-02	1.8556E-02	
	683	1.5E-06	1.7545E-02	1.9142E-02	
	684	1.5E-05	1.7970E-02	1.9520E-02	
	685	3.4E-05	1.8720E-02	1.9563E-02	
	686	4.0E-08	1.8168E-02	1.9913E-02	
	687	3.0E-14	1.7799E-02	2.0155E-02	
	688	2.0E-09	1.7321E-02	1.9254E-02	
	689	5.1E-10	1.7542E-02	1.9294E-02	
	690	1.6E-12	1.8272E-02	2.0239E-02	
	691	2.1E-17	1.9716E-02	2.2353E-02	
	692	3.7E-24	2.1352E-02	2.4469E-02	
	693	1.2E-36	2.3384E-02	2.6366E-02	
	694	1.2E-40	2.5100E-02	2.8097E-02	
	695	7.4E-45	2.6811E-02	3.0024E-02	
	696	8.4E-52	2.8946E-02	3.2289E-02	
	697	1.5E-58	3.0691E-02	3.5063E-02	
	698	1.3E-79	3.2663E-02	3.8291E-02	
	699	6.3E-83	3.4471E-02	4.0210E-02	
	700	1.4E-97	3.7695E-02	4.2988E-02	
	701	2.7E-126	3.9822E-02	4.6032E-02	
	702	3.7E-128	4.1373E-02	4.8330E-02	
	703	2.9E-106	4.3002E-02	5.0221E-02	
	704	8.8E-117	4.4824E-02	5.3168E-02	
	705	1.2E-141	4.6397E-02	5.6542E-02	
	706	7.0E-169	4.8552E-02	5.9839E-02	
	707	5.6E-173	5.0604E-02	6.1988E-02	
	708	2.2E-161	5.1895E-02	6.2775E-02	
	709	2.8E-142	5.3440E-02	6.4157E-02	
	710	1.7E-142	5.5272E-02	6.6802E-02	
	711	2.2E-187	5.7129E-02	6.9590E-02	
	712	2.9E-207	5.7895E-02	7.0674E-02	
	713	5.5E-193	5.9088E-02	7.0886E-02	
	714	8.0E-179	5.7745E-02	7.1386E-02	
	715	6.8E-185	5.7342E-02	7.2453E-02	
	716	1.1E-204	5.7935E-02	7.2380E-02	
	717	4.5E-183	5.6241E-02	7.0703E-02	
	718	1.4E-179	5.1175E-02	6.5920E-02	
	719	4.8E-180	4.8066E-02	6.1016E-02	
	720	1.9E-166	4.5448E-02	5.7158E-02	
	721	7.3E-193	4.5045E-02	5.7635E-02	
	722	5.7E-210	4.8049E-02	6.1212E-02	
	723	2.2E-193	4.9819E-02	6.3681E-02	
	724	2.8E-189	4.9258E-02	6.2972E-02	
	725	2.8E-187	4.7154E-02	5.9866E-02	
	726	6.3E-191	4.4767E-02	5.6611E-02	
	727	7.4E-197	4.3968E-02	5.5824E-02	
	728	6.0E-210	4.4285E-02	5.6024E-02	
	729	1.3E-193	4.4549E-02	5.5849E-02	

730	2.1E-185	4.2878E-02	5.5223E-02	
731	1.7E-157	4.2046E-02	5.3705E-02	
732	2.7E-155	4.2398E-02	5.3308E-02	
733	1.9E-156	4.2443E-02	5.4128E-02	
734	2.6E-143	4.2089E-02	5.4143E-02	
735	2.7E-147	4.2040E-02	5.4153E-02	
736	9.7E-148	4.1366E-02	5.3576E-02	
737	3.5E-175	4.0067E-02	5.2412E-02	
738	5.5E-182	4.0076E-02	5.1546E-02	
739	1.6E-154	4.0314E-02	5.0709E-02	
740	3.9E-144	4.0017E-02	5.0175E-02	
741	5.4E-134	3.9257E-02	4.8996E-02	
742	4.6E-143	3.8011E-02	4.8595E-02	
743	8.9E-140	3.7677E-02	4.8656E-02	
744	1.2E-132	3.8677E-02	4.8491E-02	
745	9.5E-147	3.9034E-02	4.8752E-02	
746	1.0E-156	3.8110E-02	4.7798E-02	
747	8.0E-151	3.7353E-02	4.7491E-02	
748	7.7E-148	3.6315E-02	4.8152E-02	
749	1.1E-144	3.5764E-02	4.7095E-02	
750	3.8E-70	3.5840E-02	4.6016E-02	

.

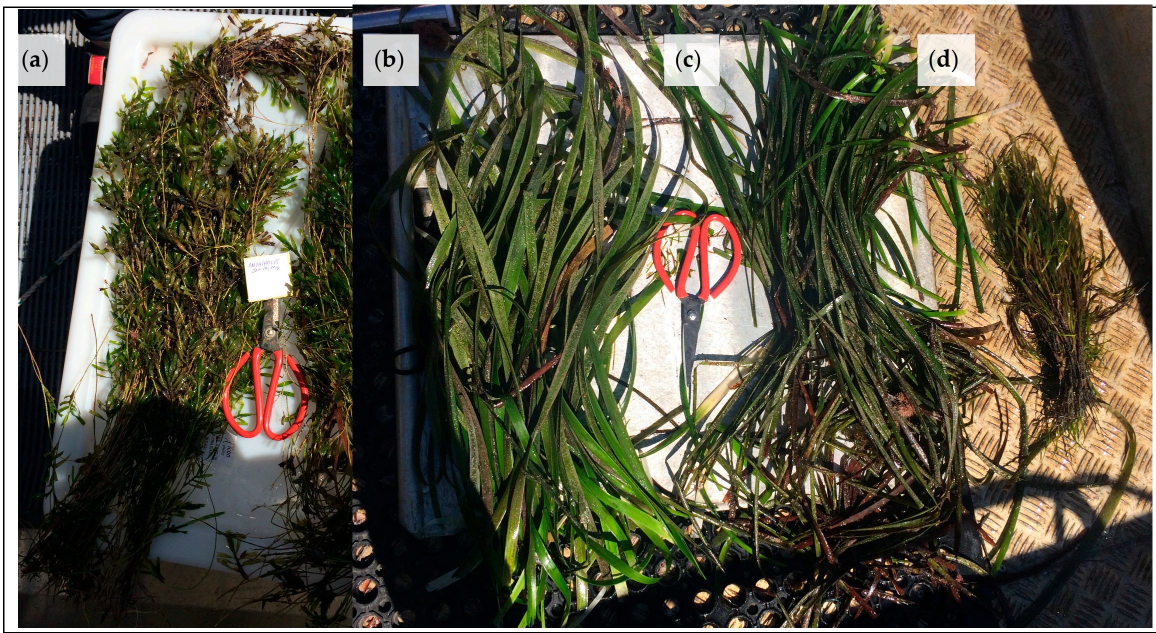

Supplementary 3. Leaf morphology of **(a)** *Amphibolis*; **(b)** *Posidonia* leaf blades; **(c)** *Posidonia* leaf sheath; and **(d)** *Heterozostera* seagrass samples collected in the field. Scissors for scale.
